# Symmetrical Heterocyclic Cage Skeleton: Synthesis, Urease Inhibition Activity, Kinetic Mechanistic Insight, and Molecular Docking Analyses

**DOI:** 10.3390/molecules24020312

**Published:** 2019-01-16

**Authors:** Muhammad Hanif, Fariha Kanwal, Muhammad Rafiq, Mubashir Hassan, Muhammad Mustaqeem, Sung-Yum Seo, Yunlong Zhang, Changrui Lu, Ting Chen, Muhammad Saleem

**Affiliations:** 1 State Key Laboratory for Modification of Chemicals Fibers and Polymer Materials, College of Chemistry, Chemical Engineering and Biotechnology, Donghua University, Shanghai 201620, China; han_qau@yahoo.com (M.H.); farihakaanwal@gmail.com (F.K.); 416033@mail.dhu.edu.cn (Y.Z.); crlu@dhu.edu.cn (C.L.); 2 Department of Chemistry, GC University Faisalabad, Sub campus Layyah 31200, Pakistan; 3 Department of Physiology and Biochemistry, Cholistan University of Veterinary and Animal Sciences, Bahawalpur 63100, Pakistan; rafiqknu@gmail.com; 4 College of Natural Science, Department of Biology, Kongju National University, Gongju, Chungcheongnam 32588, Korea; mubashirhassan_gcul@yahoo.com (M.H.); dnalove@kongju.ac.kr (S.-Y.S.); 5 Department of Chemistry, University of Sargodha, Sub-campus Bhakkar 30000, Pakistan; muhammad.mustaqeem@uos.edu.pk

**Keywords:** heterocyclic cage, urease inhibition, metal complexes, lineweaver–burk plot, molecular docking

## Abstract

The present study focuses on the design and synthesis of a cage-like organic skeleton containing two triazole rings jointed via imine linkage. These molecules can act as urease inhibitors. The in-vitro urease inhibition screening results showed that the combination of the two triazole skeleton in the cage-like morphology exhibited comparable urease inhibition activity to that of the reference thiourea while the metallic complexation, especially with copper, nickel, and palladium, showed excellent activity results with IC_50_ values of 0.94 ± 0.13, 3.71 ± 0.61, and 7.64 ± 1.21 (**3a**–**c**), and 1.20 ± 0.52, 3.93 ± 0.45, and 12.87 ± 2.11 µM (**4a**–**c**). However, the rest of compounds among the targeted series exhibited a low to moderate enzyme inhibition potential. To better understand the compounds’ underlying mechanisms of the inhibitory effect (**3a** and **4a**) and their most active metal complexes (**3b** and **4b**), we performed an enzymatic kinetic analysis using the Lineweaver–Burk plot in the presence of different concentrations of inhibitors to represent the non-competitive inhibition nature of the compounds, **3a**, **4a**, and **4b**, while mixed type inhibition was represented by the compound, **3b**. Moreover, molecular docking confirmed the binding interactive behavior of **3a** within the active site of the target protein.

## 1. Introduction

The development of competitive and non-competitive enzyme inhibitors based on a metalloskeleton has attracted wide interest of the scientific community because metal ions are found in the active sites of a large number of metalloproteins, such as hemocyanin, and also in metalloenzymes, like ureases, tyrosinase, laccase, and ascorbate oxidase [1,2,3,4,5,6,7,8]. The enzyme inhibition by the metallocompouds is due to the reaction of the organic skeleton as well as the transition metal ions with the sulfhydryl group in the active center of the enzyme [9]. Urease action results in an elevation of pH, which exerts harmful effects where it occurs whether in soil or inside the human body [10]. 

Urease inhibitors contain two general classifications, including pure organic based compounds and organic compounds, in ligation with transition metals, as some transition metals themselves possess a slight urease inhibition potential [11]. The literature survey showed excellent urease inhibition potential for candidates exhibiting hydroxamic acids, phosphoramides, and thiols’ skeleton [12]. Compounds with thiol functional groups inhibit urease competitively in their thiolate anion form, R-S^−^ [13]. Imine derivatives of organic compounds have been extensively employed as ligands for several ions, and have been utilized as pigments, catalysts, drugs, and polymer stabilizers [14]. The interested drug candidate can be designed and synthesized via a Schiff base skeleton either through insilico/bioinformatics study or by synthesizing analogues that are structurally close to the reference drug. Some of urease inhibitors cannot be used in vivo because of their toxicity or instability. Thus, seeking novel urease inhibitors with good bioavailability and low toxicity is our main focus [15]. Here, we report the synthesis and urease inhibition evaluation of metal complexes of substituted triazole based Schiff base ligands containing thiols and alcoholic moiety connected to a triazole backbone. 

## 2. Results and Discussion

### 2.1. Synthesis of Schiff Base Ligands ***1*** and ***2***

4-Methoxyphenylacetic acid was taken as starting material and then esterified into their aralkylethanoate in the presence of a catalytic amount of sulfuric acid. First, thin layer chromatographic analysis confirmed the ester formation. The acid spot remained at the bottom due to acidic protons in the hydrogen bonding with silica gel while that of ester traveled along with the direction of the solvent front. Secondly, FT-IR spectral measurement by the absence of a hydroxyl signal in the range of 3400–2500 cm^−1^ further confirmed the esterification reaction. The resulting esters were then converted to their corresponding acid hydrazides by the treatment with hydrazine hydrate in the presence of ethanol as the solvent. The new broad band signal in the range of 3342, 3287, and 3158 cm^−1^ corresponds to primary and secondary amino group stretching vibrations. Moreover, the resonance effect due to amide functionality exerts partial single bond characters in the carbonyl double bond compared to that of ester, shifting the carbonyl group signal from 1711 to 1674 cm^−1^. The emergence of signals at 1628 and 1610 cm^−1^ confirms imines’ synthesis due to the C=N stretching vibration. 

### 2.2. Ligand-Metals Complexation

**Scheme 1:**
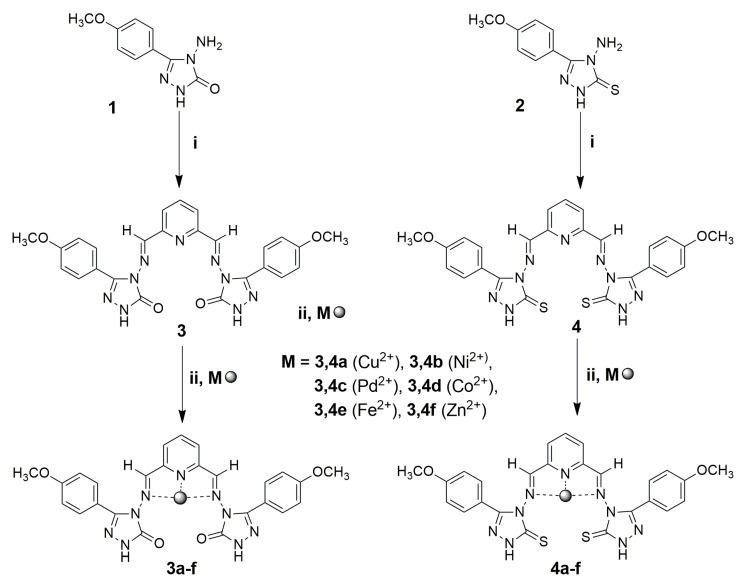
Synthesis of target molecule **3a-f** and **4a-f**; reagents and conditions: (i) Pyridine-2,6-dicarbaldehyde, glacial acetic acid (3–4 drops), absolute methanol, reflux 8 h; (ii) chloride salts of transition metals, ligands (**3** and **4**), methanol, reflux 2–3 h.

The ligand-metal complexation causes a shift in the physical appearances of the ligand before and after metal-complexation, corroborated by differences in their melting points. Further, FT-IR analysis showed that both ligands **3** and **4** contain the labile protons, which can stay inside the triazole ring nitrogen atom as well as outside the ring to the oxygen in molecule **3** and sulfur in molecule **4**. This protonic shift leads to the existence of the ligand in the tautomeric conformation. Among the tautomers, the keto tautomer seems more stable in comparison to its enol form and the same for compounds exhibiting thiol-thione tautomerism. Therefore, both ligands preferably stayed in the keto (in case of ligand **3**) and thione (in case of ligand **4**) conformation as indicated in their FT-IR absorption spectral analysis due to the appearance of signals at 1680 and 1272 cm^−1^ for ligands **3** and **4**, respectively. The spectral position for both these ligands shifts to lower frequencies after complexation, evidence for these molecular sites’ involvement during the ligation with metals. Moreover, complete disappearance of the signal at 2500 cm^−1^ in ligand **4** after metal complexation further confirms sulfur’s participation in metal binding. The similar disappearance of ligand **3’s** broad peak at 3468 cm^−1^ after metal chelation represents the interaction of undersigned oxygen with the transition metal (Table 1). The schematic representations of the synthetic route adopted to obtain the target molecule (**3a-f** and **4a-f**) are given in Scheme 1. The detailed synthetic procedure adopted for the accomplishment of precursors **1** and **2** is inserted in the supporting information. 

### 2.3. Optical Analysis

Ligand synthesis and their ligation with several metals were determined by recording their photophysical parameters via absorption spectral analysis. Ligand **3** showed two absorption bands at 240 and 304 nm while ligand **4** exhibited the maximum absorption at 247 and 292 nm, respectively. Several heteroatoms in the ligands’ red-shifts the absorption wavelength while the pi-electronic transition blue-shifts the wavelength due to a typical longer energy gap between the ground and excited state for labile π-electrons systems. Complexation leads to almost negligible variation in the absorption maxima position due to π-electrons systems. However, ligation caused a red-shift due to non-bonding electrons, which indicates the affiliation of lone pair electrons of heteroatoms toward metals (Table 2).

### 2.4. Ligand Safety Profiles

The ligand safety was assessed after 4 and 24 h treatment to the cells by MTT [3-(4,5-dimethylthiazol-2-yl)-2,5-diphenyltetrazolium bromide] assay. The results showed no toxicity for L-929 cells at 50 μM ligand concentrations. The safe profile of the ligand towards the living cells might open the ligands’ possibility as bioimaging probes. The safe nature of the triazole skeleton warrants further characterization of the compound. 

### 2.5. Bio-Evaluation

#### Urease Inhibition Activity

We tested our synthesized compounds, **3a-f** and **4a-f**, for their inhibitory effects on urease. Besides current urease inhibitors [16], we aimed to investigate the effect of the two triazole ring structure mutually coupled with each other toward urease inhibition as well as to understand the effect of metal complexation toward the bio-profile of the designed nucleus. The in-vitro screening results showed that the combination of the two triazole skeleton in the cage like morphology exhibited comparable urease inhibition potential to that of the reference. The metallic chelation with copper, nickel, and palladium showed inhibition with IC_50_ values of 0.94 ± 0.13, 3.71 ± 0.61, and 7.64 ± 1.21 (**3a**–**c**) and 1.20 ± 0.52, 3.93 ± 0.45, and 12.87 ± 2.11 µM (**4a**–**c**). Copper chelation produced a maximum inhibitory profile possibly due to interactions with the tested protein. The overall bioprofile of oxygen containing the triazole cage and their metal complexes exhibited higher inhibition than the sulfur containing triazole cage and their corresponding metal complexes. In general, iron and zinc compounds displayed the least inhibition, while the cobalt complex fell in the middle (Table 3). 

### 2.6. Mechanism Underlying Inhibitory Effect of Compounds ***3a***, ***4a***, ***3b***, and ***4b***

**Figure 1:**
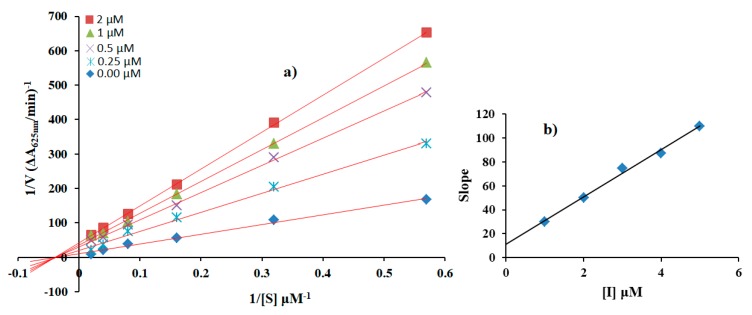
Kinetic analysis results for target molecule **3a**. (**a**) Lineweaver-Burk plots for the inhibition of urease in the presence of compound **3a**; concentrations of **3a** of 0, 0.25, 0.5, 1, and 2 µM, respectively. Substrate urea concentrations were 1.57, 3.12, 6.25, 12.5, 25, and 50 µM, used respectively; (**b**) The secondary replot of the Lineweaver-Burk plot, slope vs. various concentrations of **3a**.

**Figure 2:**
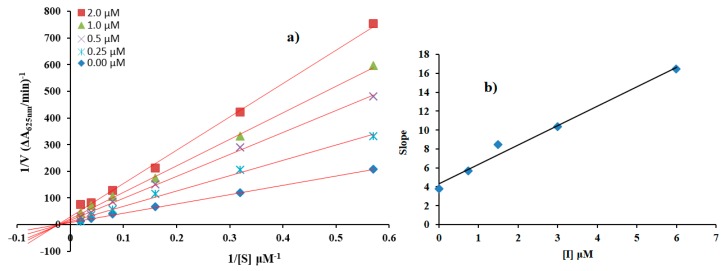
Kinetic analysis results for target molecule **4a**. (**a**) Lineweaver-Burk plots for the inhibition of urease in the presence of compound **4a**. Concentrations of **4a** of 0, 0.75, 1.5, 3, and 6 µM, respectively. Substrate urea concentrations were 1.57, 3.12, 6.25, 12.5, 25, and 50 µM, used respectively; (**b**) The secondary replot of the Lineweaver-Burk plot, slope vs. various concentrations of **4a**.

**Figure 3:**
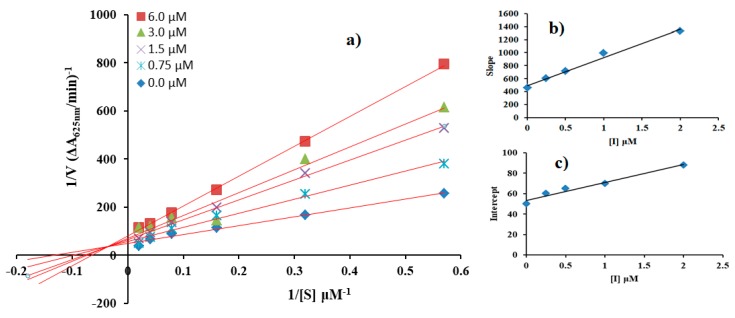
Kinetic analysis results for target molecule **3b**. (**a**) Double reciprocal Lineweaver-Burk plots for the inhibition of Jack bean urease in the presence of compound **3b**. Concentrations of **3b** were 0, 0.25, 0.5, 1, and 2 µM, respectively. Substrate urea concentrations were 1.57, 3.12, 6.25, 12.5, 25, and 50 µM, used respectively; (**b**) The secondary replot of the Lineweaver-Burk plot, slope vs. various concentrations of **3b**; (**c**) The secondary replot of the Lineweaver-Burk plot, Intercept vs. various concentrations **3b**.

**Figure 4:**
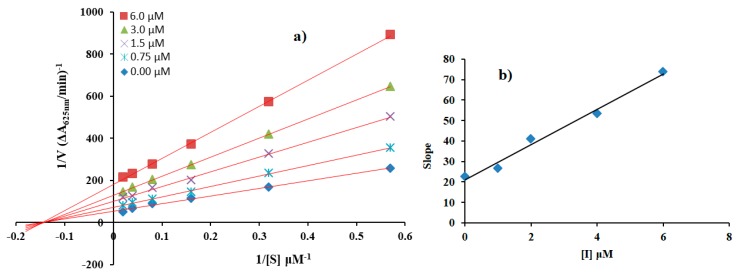
Kinetic analysis results for target molecule **4b**. (**a**) Lineweaver-Burk plots for the inhibition of urease in the presence of compound **4b**; concentrations of **4b** were used as 0, 1, 2, 4, and 6 µM, respectively. Substrate (urea) concentrations, 1.57, 3.12, 6.25, 12.5, 25, and 50 µM, were used, respectively; (**b**) The secondary replot of the Lineweaver-Burk plot, slope vs. various concentrations of **4b**.

Next, we picked compound **3a**, **4a**, **3b**, and **4b** for further tests against the urease enzyme to investigate the underlying mechanism. We plotted 1/V versus 1/[S] in the presence of different concentrations of inhibitors, **3a**, **4a**, **3b**, and **4b**, using the Lineweaver-Burke plot to study the resulting enzyme kinetics (Figure 1, Figure 2, Figure 3 and Figure 4a,b) show the inhibition constants (*Ki*) calculated from the Lineweaver-Burke plots. The results showed that compounds **3a**, **4a**, and **4b** behaved as a non-competitive inhibitor (Figure 1, Figure 2 and Figure 4a), which means that 1/V_max_ increased while K_m_ remained constant under increasing concentrations of compounds **3a**, **4a**, and **4b**, respectively. This behavior indicated that compounds **3a**, **4a**, and **4b** inhibit urease non-competitively to form enzyme inhibitor (EI) complex [16]. Specifically, data from compound **3b**, with an increasing the concentration of the substrate (urea), all intersected within the second quadrant. This result showed that *V_max_* decreased with increasing *K_m_* with increasing concentrations of **3b**. This behavior indicated that compound **3b** is a mixed type inhibitor with respect to the substrate, urea, with a *Ki* value of 1.2 µM and a *Ki'* value of 3.0 µM as shown in Figure 3b,c. The results of the kinetic constants and inhibition constants are summarized in Table 4. The kinetic data is graphically explained in Figure 1, Figure 2, Figure 3 and Figure 4.

### 2.7. Structural Assessment of Jack Bean Urease

**Figure 5:**
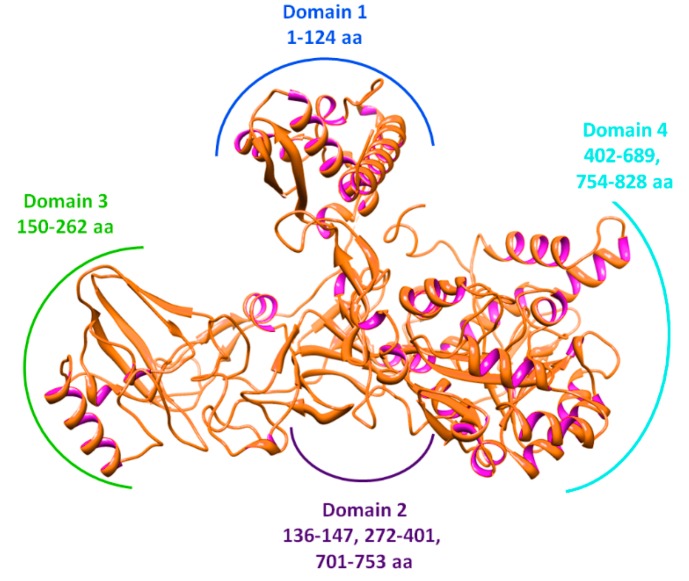
Crystal structure of jack bean urease.

The metal-containing jack bean urease contains four unique structural domains (Figure 5) [17]. Two nickel atoms coordinate key structural interactions in domain four. Structural data revealed that copper atoms can directly interact with His545, His519, His409, His407, and Asp633 within the active binding pocket of jack bean urease. The VADAR analysis showed that the protein contains 27% helices, 31% β sheets, and 41% coils, while the Ramachandran plot indicated that 97.5% of residues fall in favored regions. The Ramachandran graph is mentioned in the supplementary data.

### 2.8. Docking Shows Binding Energy and Conformation

**Figure 6:**
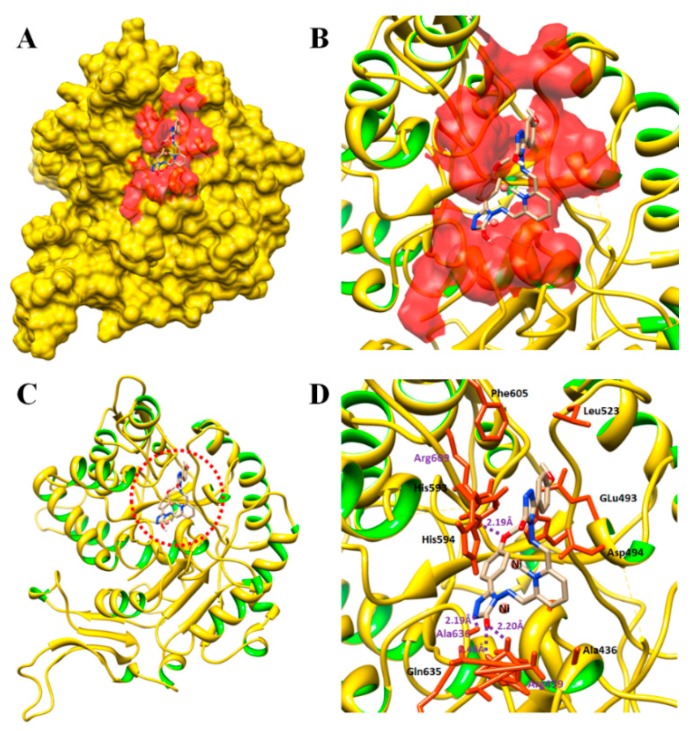
Docking results of **3a** complex with jack bean urease. (**A**) the protein molecule is rendered as the yellow surface, whereas the binding pocket is highlighted in maroon. (**B**) The closer view of the docking complex with the **3a** ligand. Two nickel atoms are labeled in brown. (**C**) The ribbon format protein structure with the interactive ligand. (**D**) The interactive residues are highlighted in the dark brown color and the ligand (**3a**) is justified in grey color with different moieties’ colors. The purple lines represent hydrogen bonding and the distance mentioned in angstrom (Å).

Based on in vitro results, we chose **3a** for binding conformation inside the active site of jack bean urease. Docking and fitting (**3a**) calculated a binding energy value of −10.40 kcal/mol. The **3a**-docked complex showed that compound **3a** was enclosed in the active site of the jack bean urease. Compound **3a** formed four active hydrogen bonds with the protein active site. The carbonyl oxygen atom on the triazole ring was H-bonds with Arg439 residue with bond lengths of 2.20 and 2.46 Å, respectively. Similarly, the triazole N2 hydrogen likely interacted with Ala636 through hydrogen bonding, having a bond length of 2.19 Å. Moreover, the carbonyl oxygen formed another hydrogen bond with Arg609 with a bond length of 2.19 Å (Figure 6). The detailed interactive behavior of **3a** and urease showed that in Arg609 bonding, the oxygen atom of **3a** acts as an acceptor whereas the hydrogen atom of Arg609 behave as a donor atom. Similarly, the oxygen and nitrogen atoms act as acceptors and donor atoms in Ala636 bonding, respectively. The significant binding was observed with Arg439 at two different positions against **3a**, the oxygen atom of compound **3a** behaves as an acceptor while the hydrogen atoms of Arg439 behaves as donor atoms in both bonding. The literature also shows similar results with other urease inhibitors, which corroborates our docking results [18,19,20]. These combined results indicate that the **3a** compound may be a potent inhibitor of jack bean urease. Docking results of **3a** complexed with jack bean urease are exhibited in Figure 6. 

## 3. Materials and Methods

### 3.1. Substrate and Reagents

The 4-methoxybenzoic acid, pyridine-2,6-dicarbaldehyde, phosphorous oxychloride, carbohydrazide, isocyanate and isothiocyanates, Hydrazine hydrate (80%), TEA, CS_2_, KOH, sodium hydrogen carbonate, and glacial acetic acid were purchased from Sigma-Aldrich, Darmstadt, Germany. The chloride salts of the transition metals were obtained from Aldrich and Alfa Aesar. Ethanol, methanol, chloroform, deionized water, acetonitrile, dimethyl sulfoxide, petroleum ether, ethyl acetate, n-hexane, toluene (Samchun Chemicals, Seoul, Korea), H_2_SO_4_, acetic acid, and HCl (Jin Chemical and Pharmaceutical Co. Ltd., Seoul, Korea) were used in this experiment. Urease from jack bean (EC 3.5.1.5), Thio-urea, sodium nitroprusside, and active chloride were purchased from Sigma (St. Louis, MO, USA). Stock solutions of the reducing substrates were prepared in phosphate buffer (20 mM, pH 6.8). 

### 3.2. Instrumentations

The reaction progress was monitored by thin layer chromatography (TLC), and the R*_f_* values were determined with pre-coated silica gel aluminum plates, Kieselgel 60 F_254_ from Merck (Darmstadt, Germany). TLC plates were visualized under a UV lamp (VL–4 LC, Collégien, France). The melting points were determined on a Fisher Scientific (Waltham, MA, USA) melting point apparatus. The FT-IR spectra were recorded in KBr pellets on a Shimadzu FTIR–8400S spectrometer (Kyoto, Japan). Proton and carbon nuclear magnetic resonance (^1^H-NMR and ^13^C-NMR) spectra were recorded on a Bruker Avance 400 MHz spectrometer with TMS as an internal standard. The chemical shifts are reported as *δ* values (ppm) downfield from the internal tetramethylsilane of the indicated organic solution. Peak multiplicities are expressed as follows: S, singlet; d, doublet; and m, multiplet. Mass spectra were recorded on the AB SCIEX Co. 4000 QTRAP LC/MS/MS System. The UV-visible absorption measurements were carried out using [SCINCO] UV-Vis Spectrophotometer “S-3100” (SCINCO, Seoul, Korea). Abbreviations are as follows: CD_3_OD, deuterated methanol; DMSO–*d*_6_, dimethyl sulfoxide-*d*_6_, and FT-IR spectroscopy, Fourier transform infrared spectroscopy. 

### 3.3. Synthesis of 4-amino-3-(4-methoxyphenyl)-1H-1,2,4-triazol-5(4H)-one (***1*** and ***2***)

The 4-methoxybenzoyl chloride was synthesized by reacting 4-methoxybenzoic acid (1 mmol) in the presence of 1,2–dichloroethane (12 mL) solvent and phosphorous oxychloride (0.4 mL) as the chlorinating agent under reflux for 3 h. Then, the resulting solution was cooled to room temperature, and the solvent was removed under reduced pressure. The 4-methoxybenzoyl chloride and carbohydrazide were separately dissolved in the dichloromethane and mixed together slowly at low temperature with continuous stirring followed by reflux for 3 h. The reaction progress was continuously monitored after every 30 min by using aluminium pre-coated silica gel TLC plates. The product was extracted by using dichloromethane and anhydrous magnesium sulfate and purified by column chromatography employing a dichloromethane:hexane solvent system. Compound **2** was prepared according to our previously reported procedure [21]. 

### 3.4. Synthesis of Schiff Base Derivative ***3***, ***4*** and Their Metal Complexes

The Schiff base derivative **3** and **4** were synthesized following our previously reported methods [21]. The metal complexes 3,4a-f were synthesized by refluxing an equimolar quantity of the respective metal salts with the Schiff base ligands **3** and **4**, utilizing ethanol as the solvent. The formation of the transition metal complexes was initially indicated by the TLC analysis as well as by visual detection due to a colorimetric change in the reaction mixture. Further characterization was performed by the FT-IR, UV-visible, and fluorescence spectroscopic analysis. Anal. Calcd. for target: **3**, C_25_H_21_N_9_O_4_: C, 58.70; H, 4.14; N, 24.65; found: C, 58.66; H, 4.11; N, 24.21. for target: **4**, C_25_H_21_N_9_O_2_S_2_: C, 55.23; H, 3.89; N, 23.19, found: C, 55.18; H, 3.85; N, 23.16. 

### 3.5. General Procedure for Spectroscopic Measurement

The ligands (**3** and **4**) and their metal complexes (**3-f** and **4a-f**) stock solution (1 mM) were prepared by dissolving 5.11 and 5.43 mg of desired compounds in THF (total volume 10 mL). For spectroscopic measurements, the test solution of 3 mL was prepared with 2.34 mL of THF, 90 μL of ligand stock solution, 0.3 mL of buffer solution (EtOH/PBS buffer saline, 100 mM), and 90 μL of ionic stock solution. The resulting solutions were mixed before measurement and the final volume was fixed as 3 mL for UV-visible and fluorescent studies. All recognition studies were performed at 25 °C while the samples were shaken to ensure solution uniformity prior to spectrum recording. The limit of detection and association constants for the probe on metal binding were found by the absorption titration experiments while the average Stern-Volmer quenching constants for the probe towards cobalt, mercury, and copper were calculated by fitting the emission titration results to the Stern-Volmer plot [22,23,24].

### 3.6. General Procedure for MTT Assay

MTT assay was done following the reported procedure [25]. Briefly, the cells were incubated with ligand and their metal complexes (**3** and **4**; **3a-f** and **4a-f**) (60 μM) for 24 h. Then, cells were washed with phosphate buffered saline (PBS), and incubated with Dulbecco’s Modified Eagle’s medium (DMEM medium, 200 μL/well) containing 50 μL of MTT [3-(4,5-dimethylthiazol-2-yl)-2,5-diphenyltetrazolium bromide, 5 mg/mL] solution. Following 2 h of incubation at 37 °C, growth medium was removed gently from the plate and 200 μL/well dimethyl sulfoxide was added to solubilize the produced purple formazan crystals. Later, the absorbance for each well was measured at 570 nm using microplate spectrophotometer systems (BioTek, synergy HT) and results were calculated in percentage with respect to the untreated sample called the control. The same procedure was adopted for all the compounds and their metal complexes.

### 3.7. General Procedure for Urease Inhibition Assay

The urease activity was determined by measuring the amount of ammonia produced with the indophenols method previously described [26,27]. The reaction mixtures, comprising 20 µL of enzyme (jack bean urease, 5 U/mL) and 20 μL of test compounds in 50 μL buffer (100 mM urea, 0.01 M K_2_HPO_4_, 1 mM EDTA, and 0.01 M LiCl_2_, pH 8.2), were incubated for 30 min at 37 °C in a 96-well plate. Briefly, 50 μL each of phenol reagents (1%, *w/v* phenol and 0.005%, *w/v* sodium nitroprusside) and 50 μL of alkali reagent (0.5%, *w/v* NaOH and 0.1% active chloride NaOCl) were added to each well. The absorbance at 625 nm was measured after 10 min, using a microplate reader (OPTI Max, Tunable). All reactions were performed in triplicate. The urease inhibition activities were calculated according to the following formula:

Urease inhibition activity (%) = (ODcontrol − ODsample × 100)/ODcontrol

where OD_control_ and OD_sample_ represents the optical densities in the absence and presence of the sample, respectively. Thiourea was used as the standard inhibitor for urease.

### 3.8. Computational Methodology

#### Retrieval of Jack Bean Urease

The three-dimensional protein structure of jack bean urease (*C. ensiformis*) was accessed from the Protein Data Bank (PDB) (www.rcsb.org) with PDBID 4H9M. The selected protein structure was minimized by employing UCSF Chimera 1.10.1 [28]. Molprobity server [29] was used to predict the Ramachandran plot and values of the target structure [30]. The protein architecture and statistical percentage values of receptor proteins, helices, beta-sheets, coils, and turns were predicted from the online server, VADAR 1.8 [31].

### 3.9. Molecular Docking

Based on the in vitro results, **3a** was selected for the docking experiment to check the binding against the target protein. Before the docking experiment, the synthesized ligand (**3a**) was sketched in the ACD/ChemSketch tool and accessed in the mol format. Furthermore, the UCSF Chimera 1.10.1 tool was employed for energy minimization of **3a** having default parameters. The steepest descent steps were adjusted to 100 with a step size of 0.02 (Å), conjugate gradient steps also fixed to 100 with a step size of 0.02 (Å), and the update interval was retrained at 10. Finally, Gasteiger charges were added using Dock Prep in the ligand structure to obtain the good structure conformation. The molecular docking experiment was run through the PyRx virtual screening tool with the AutoDock VINA Wizard approach [32,33]. The grid box center values (X = 10.22, Y = 24.56 and Z = 46.18) were adjusted with default exhaustiveness value = 8 to maximize the binding conformational analysis. The generated docked complexes were evaluated based on the lowest binding energy (Kcal/mol) values and structure activity relationship (SAR) analyses. The three dimensional (3D) graphical depictions of the **3a**-docked complex were accomplished by Chimera 1.10.1.

## 4. Conclusions

In this study, we joined two triazole rings to form a series of Schiff base derivatives into a cage-like structure. Then, we evaluated their urease inhibition activity as well as their underlying mechanism for inhibition with enzyme kinetics studies. Among the tested series, copper, nickel, and palladium complexes displayed appreciable enzyme inhibition results, while the rest showed low to moderate enzyme inhibition potential. The kinetic analysis by the Lineweaver–Burk plot suggested the non-competitive inhibition nature of the compounds, **3a**, **4a**, and **4b**, while mixed type inhibition by the compound, **3b**. Further, molecular docking studies showed that **3a** interacted within the active region of jack bean urease and exhibited a good binding energy value of −10.40 kcal/mol. These combined results showed that synthesized molecules can serve as structural templates in the design of novel drugs against the urease enzyme.

## Figures and Tables

**Table 1:** FT-IR spectroscopic data for ligands **3** and **4** before and after complexation.

S. No.	Compounds	-N-H	C-H	C=N	C=C	C=S	C=O	C-O-C
**1**	ligand (**3**)	3519–3738	3138–2949	1638–1620	1511–1400	--	1680	1176
**2**	**3a** (ligand+Cu^2+^)	3491–3370	3190–2901	1610, 1584	1521–1411	--	1640	1171
**3**	**3b** (ligand+Ni^2+^)	3481–3401	3200–2911	1612, 1588	1505–1431	--	1638	1176
**4**	**3c** (ligand+Pd^2+^)	3499–3378	3205–2933	1620, 1591	1505–1430	--	1638	1170
**5**	**3d** (ligand+Co^2+^)	3501–3391	3178–2918	1620, 1588	1508–1428	--	1640	1170
**6**	**3e** (ligand+Fe^2+^)	3511–3381	3186–2913	1618, 1601	1515–1429	--	1638	1168
**7**	**3f** (ligand+Zn^2+^)	3508–3402	3191–2939	1622, 1601	1511–1401	--	1638	1180
**8**	ligand (**4**)	3523–3390	3196–2950	1628, 1610	1504–1424	1272	--	1176
**9**	**4a** (ligand+Cu^2+^)	3505–3401	3190–2940	1618, 1602	1511–1438	1231	--	1188
**10**	**4b** (ligand+Ni^2+^)	3511–3370	3188–2911	1609, 1608	1521–1411	1241	--	1178
**11**	**4c** (ligand+Pd^2+^)	3500–3388	3201–2933	1622, 1599	1518–1421	1238	--	1178
**12**	**4d** (ligand+Co^2+^)	3511–3401	3174–2919	1613, 1584	1515–1409	1232	--	1187
**13**	**4e** (ligand+Fe^2+^)	3508–3391	3181–2909	1617, 1602	1511–1434	1240	--	1188
**14**	**4f** (ligand+Zn^2+^)	3508–3400	3199–2919	1611, 1602	1505–1419	1236	--	1175

**Table 2:** Physical characteristics and optical analysis results for ligands **3** and **4** before and after complexation.

S. No.	Compounds	Appearance	m.p. °C	λ_abs_ (nm) ^a^	ε 10^5^ (M^−1^cm^−1^) ^b^
**1**	ligand (**3**)	yellow powder	211–213	240, 304	6.4, 3.8
**2**	**3a** (ligand+Cu^2+^)	dark yellow	232–240	240, 361	6.4, 3.41
**3**	**3b** (ligand+Ni^2+^)	purple	241–243	240, 350	6.31, 3.84
**4**	**3c** (ligand+Pd^2+^)	barn red	240–242	241, 331	6.54, 3.58
**5**	**3d** (ligand+Co^2+^)	light green	238–240	246, 333	6.11, 3.61
**6**	**3e** (ligand+Fe^2+^)	chocolate	243–245	240, 340	6.76, 3.58
**7**	**3f** (ligand+Zn^2+^)	sage	230–232	240, 324	6.39, 3.38
**8**	ligand (**4**)	light yellow	183–185	247, 292	5.04, 2.7
**9**	**4a** (ligand+Cu^2+^)	dark yellow	236–238	247, 321	5.11, 2.78
**10**	**4b** (ligand+Ni^2+^)	purple	235–237	247, 311	5.21, 2.38
**11**	**4c** (ligand+Pd^2+^)	chili	244–246	247, 333	5.21, 2.81
**12**	**4d** (ligand+Co^2+^)	dark green	233–235	247, 338	5.08, 2.11
**13**	**4e** (ligand+Fe^2+^)	chocolate	239–241	247, 302	5.18, 2.02
**14**	**4f** (ligand+Zn^2+^)	sage	229–231	247, 333	5.18, 3.34

^a^ UV-visible absorption maxima; ^b^ Molar absorption coefficient.

**Table 3:** The inhibitory effect of synthesized compounds toward urease.

Compounds Code	Urease Inhibition IC_50_ (µM)
3	18.92 ± 1.81
3a	0.94 ± 0.13
3b	3.71 ± 0.61
3c	7.64 ± 1.21
3d	28.93 ± 3.11
3e	37.46 ± 4.23
3f	45.78 ± 5.24
4	29.33 ± 3.32
4a	1.20 ± 0.52
4b	3.93 ± 0.45
4c	12.87 ± 2.11
4d	23.72 ± 3.33
4e	58.83 ± 7.31
4f	39.89 ± 6.93
Thio-Urea	20.7 ± 0.45

**Table 4:** Kinetic analysis of compounds, **3a**, **4a**, **3b**, and **4b**.

Compounds	Dose (µM)	1/V_max_ (ΔA/Sec)	K_m_ (µM)	Inhibition Type	*Ki* (µM)	*Kiʹ* (µM)
**3a**	0.0	12	2.5	Non-competitive	0.6	---
	0.25	20	2.5			
	0.50	30	2.5			
	1.0	35	2.5			
	2.0	44	2.5			
**4a**	0.0	8	0.47	Non-competitive	2.1	---
	0.75	12	0.47			
	1.5	18	0.47			
	3.0	22	0.47			
	6.0	35	0.47			
**3b**	0.0	50	7.14	Mixed-inhibition	1.2	3.0
	0.25	60	10			
	0.5	65	12.5			
	1.0	70	14.2			
	2.0	80	16.6			
**4b**	0.0	55	0.41	Non-competitive	2.4	---
	1.0	65	0.41			
	2.0	100	0.41			
	4.0	130	0.41			
	6.0	180	0.41			

*V_max_* is the reaction velocity; *K_m_* is the Michaelis-Menten constant; *Ki* is the EI dissociation constant; *Kiʹ* is the ESI dissociation constant; ---: not determined

## Supplementary Materials

Supplementary Materials are available online.
