# Supplementary material for: Symmetrical Heterocyclic Cage Skeleton: Synthesis, Urease Inhibition Activity, Kinetic Mechanistic Insight, and Molecular Docking Analyses
Source: Molecules. 2019 Jan 16;24(2):312. doi: 10.3390/molecules24020312 (PMC6359172; doi:10.3390/molecules24020312)
Supplement: Supplementary file 1 [file molecules-24-00312-s001.pdf]

Supporting information

Symmetrical Heterocyclic Cage Skeleton: Synthesis, Urease Inhibition Activity, Kinetic Mechanistic Insight and Molecular Docking analyses

Synthesis of 4-amino-3-(4-methoxyphenyl)-1*H*-1,2,4-triazole-5(4*H*)-thione

Substituted aromatic acid was esterified by refluxing in ethanol in the presence of catalytic amount of sulfuric acid. Substituted aromatic esters were converted into their corresponding acid hydrazides by refluxing in hydrazine hydrate using ethanol. The potassium hydroxide (0.125 mol) was dissolved in dry methanol (50 ml). To the above solution, the substituted acid hydrazide (0.125 mol) was added and cooled the solution in ice. To this, carbon disulfide (0.125 mol) was added slowly with constant stirring. The solid product of potassium dithiocarbazate formed, was filtered, washed with chilled diethyl ether and used in the next step by taking in water (20 mL), mixed with hydrazine hydrate and allowed to reflux for 10-12 hours. The reaction mixture turned to green with evolution of hydrogen sulphide and finally it became homogeneous. It was then poured in crushed ice and neutralized with concentrated hydrochloric acid. The white precipitates appeared on acidic induction was filtered, washed with chilled water and recrystallized from aqueous methanol.

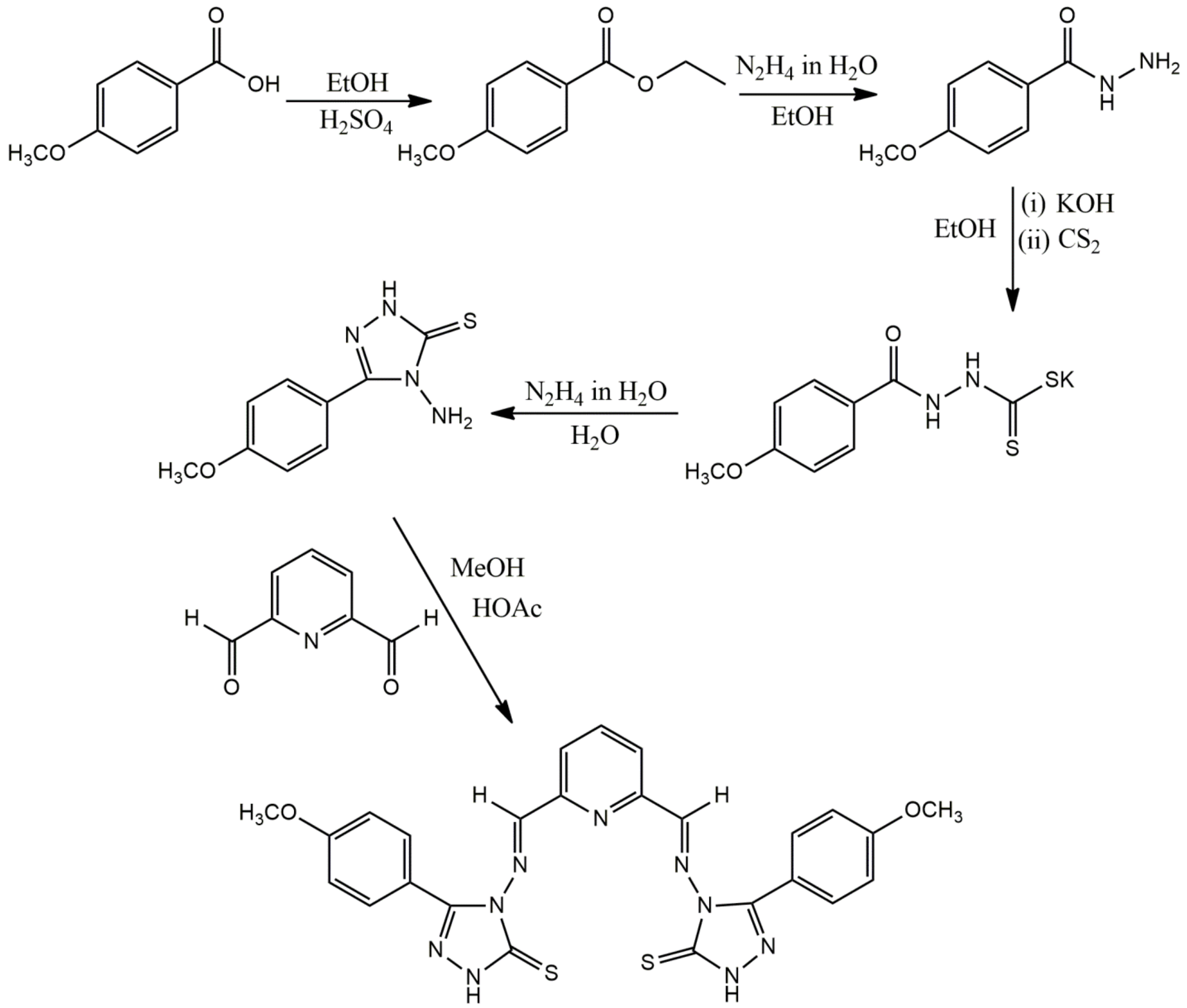

Scheme: Synthesis of 4,4'-((1E,1'E)-(pyridine-2,6-diylbis(methanylylidene))bis(azanylylidene)) bis(3-(4-methoxy phenyl)-1H-1,2,4-triazole-5(4H)-thione): Reagents and conditions: (i) Ethanol, H2SO4 (4-5 drops), reflux 3-5 h; (ii) Hydrazine hydrate, ethanol, reflux 12 h; (iii) CS2, KOH, methanol, stirring, 0 °C, 1 h; (iv) Hydrazine hydrate (80 %), water, reflux, 10-12 h; (v) 4-amino-3-(4-methoxyphenyl)-1H-1,2,4-triazole-5(4H)-thione, pyridine-2,6-dicarbaldehyde, methanol, reflux 24 h. The characterization data for the substituted hydrazide and triazole are given below.

4-Methoxybenzohydrazide

White solid; yield: 84 %; mp: 133-135 °C; R_f: 0.38 (chloroform : methanol, 9:1); FT–IR (υ /cm^{-1}): 3342, 3287 (NH_2), 3158 (NH) 3030 (sp^2 CH), 2937, 2874 (sp^3 CH), 1628 (C=O), 1554, 1507, 1486 (C=C of phenyl ring); ^1H NMR (400 M Hz, DMSO-d_6) δ 9.21 (s, 1H, NH), 7.36-7.24 (m, 2H, Ar-H), 6.97-6.84 (m, 2H, Ar-H), 4.27 (s, 2H, broad, NH_2), 3.74 (s, 3H, OCH_3); ^{13}C NMR (100 MHz, DMSO-d_6) δ168.7, 159.1, 136.5, 132.7, 129.4, 126.2, 114.5, 57.1.

4-amino-3-(4-methoxyphenyl)-1*H*-1,2,4-triazole-5(4*H*)-thione

White solid; yield: 78 %; mp: 215-217 °C; R_f: 0.23 (*n*-hexane : ethyl acetate, 7:3); IR (υ /cm^{-1}) 3277, 3181 (NH_2), 3057 (sp^2 CH), 2932, 2874 (sp^3 CH), 1597 (C=N), 1553, 1507, 1484 (C=C), 1320 (C=S); ^{1}H NMR (400 MHz, $CDCl_3$) δ 13.64 (s, 1H, NH), 7.41-7.34 (m, 2H, Ar-H), 6.98-6.85 (m, 2H, Ar-H), 5.61 (s, 2H, broad, NH_2), 3.75 (s, 3H, OCH_3); ^{13}C NMR (100 MHz, $CDCl_3$) δ 167.1, 159.5, 150.3, 135.2, 131.7, 126.1, 119.5, 114.5, 56.9.

Synthesis of 4,4'-[(1*E*,1'*E*)-{pyridine-2,6-diylbis(methanylylidene)}bis(azanylylidene)]bis(3-(4-methoxyphenyl)-1*H*-1,2,4-triazole-5(4*H*)-thione) (targeted receptor)

The substituted triazoles (0.47 g, 0.0021 mol, 2 eq) was dissolved in methanol (20 mL) and pyridine-2,6-dicarbaldehyde (0.14 g, 0.001 mol, 1 eq) was separately dissolved in methanol (20 mL) and both solution mixed together, added 4-6 drops of acetic acid as a catalyst, prior to reflux at 80 °C for 24 h. In the start of reaction, there was clear solution which turns to yellow with the evolution of precipitates with the passage of time. On complete consumption of starting materials, the precipitates were filtered to afford the target molecule. Yellow powder; mp: 183-185 °C; FT-IR (ν/cm^{-1}): 3523-3390 cm^{-1} (-NH), 3196-2950 (sp^2 C-H), 1628, 1610 (C=N), 1504-1424 (C=C), 1251 (C=S), 1176 (C-O-C); ^{1}H NMR (400 MHz, DMSO–d_6) δ 14.25 (s, 2H, NH), 10.18 (s, 2H, imine proton), 8.25-8.23 (m, 2H, aromatic proton), 8.19-8.16 (m, 1H, aromatic proton), 7.88-7.84 (m, 4H, aromatic proton), 7.12-7.08 (m, 4H, aromatic proton), 3.83 (s, 6H, OCH_3); ^{13}C NMR (100 MHz, DMSO–d_6) δ 163.1, 162.5, 161.5, 152.3, 149.5, 139.4, 130.7, 124.4, 118.0, 114.6, 67.4, 55.8; LC-MS for $C_{25}H_{21}N_9O_2S_2$ (ESI, positive mode, m/z), 545 $[M + H]^+$.

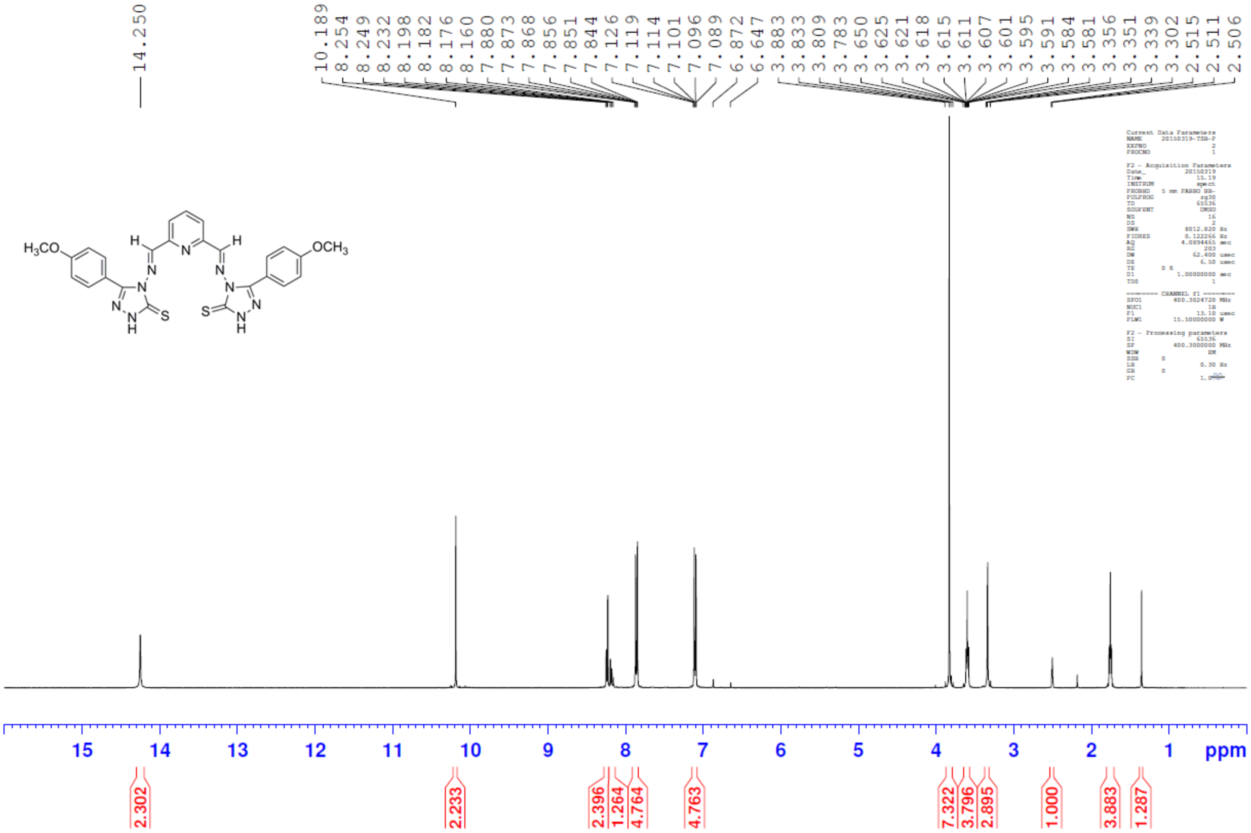

^{1}H NMR of ligand alone

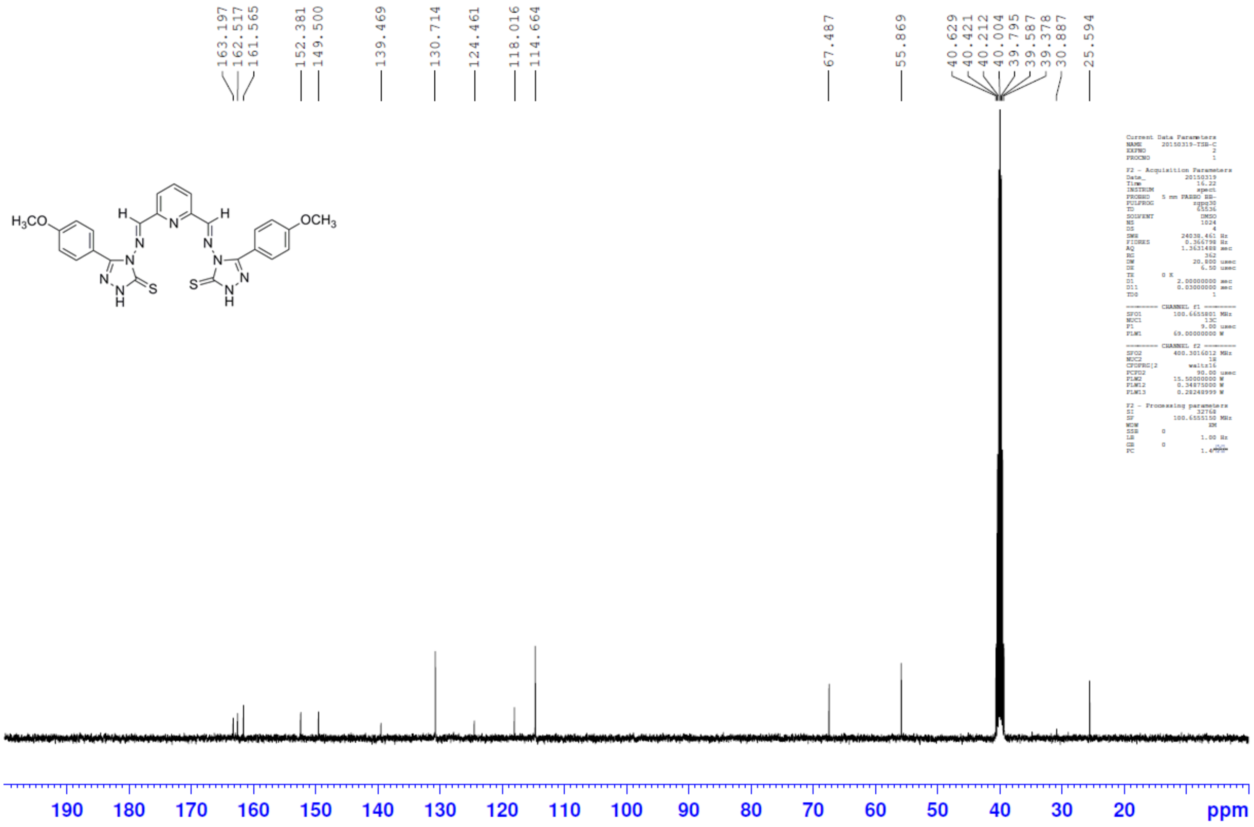

^{13}C NMR of ligand alone

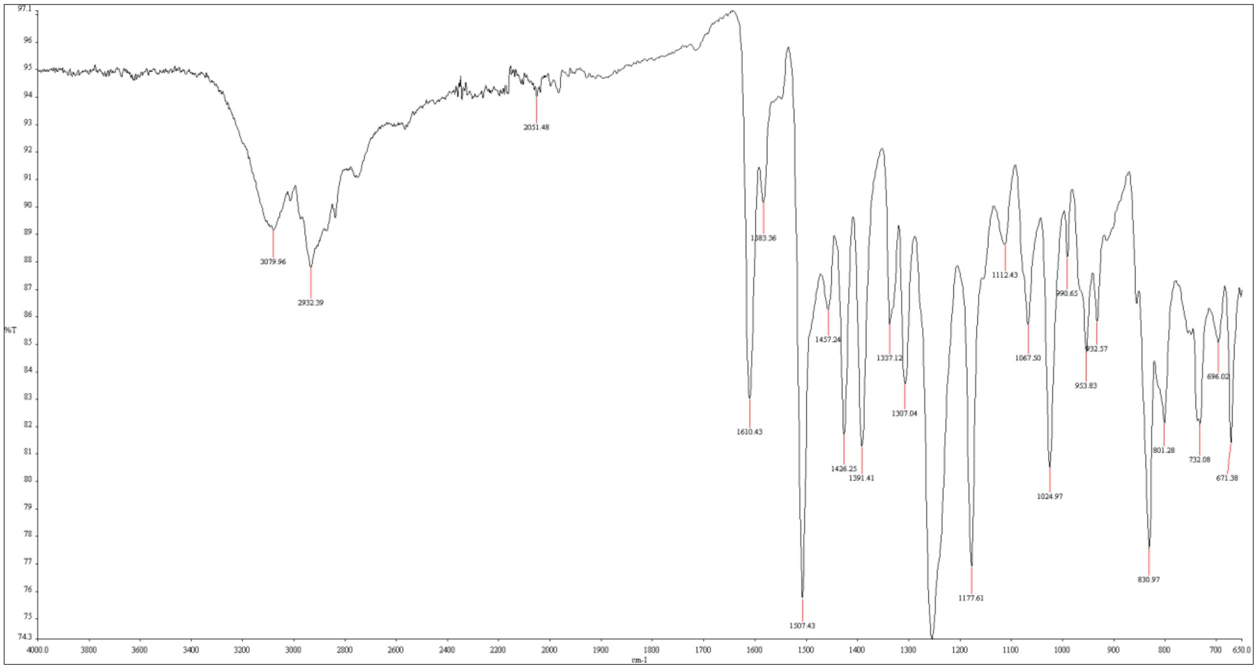

FT-IR spectrum of target molecule before complexation

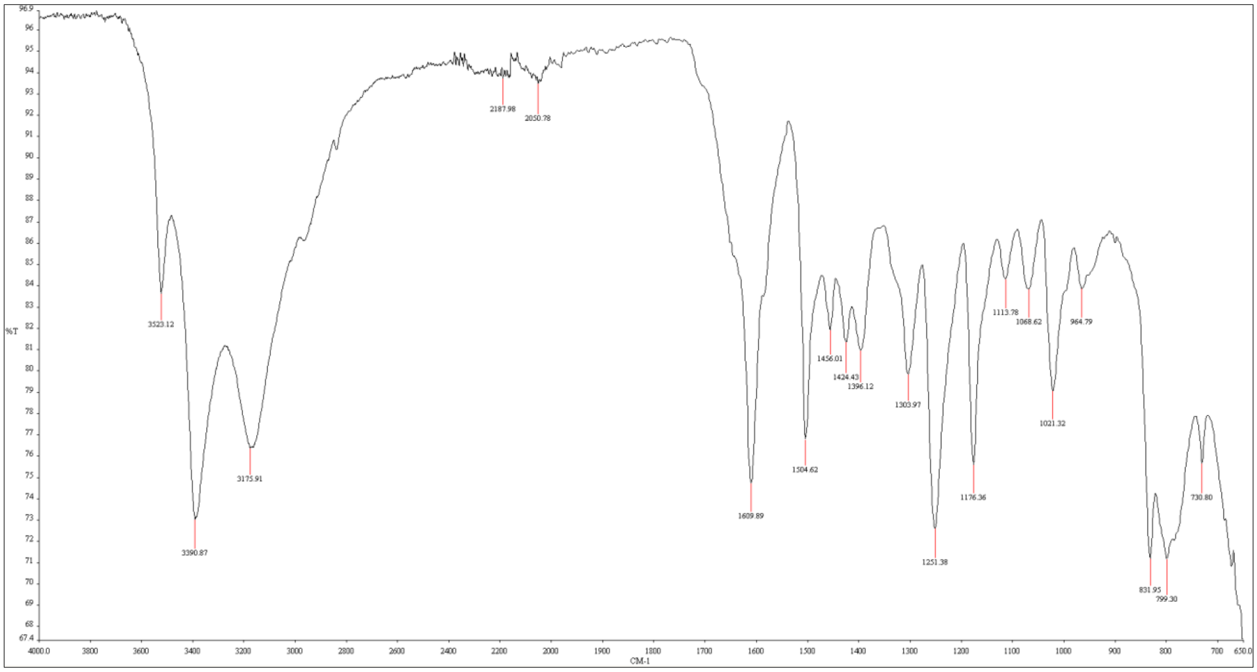

FT-IR of ligand after cobalt complexation

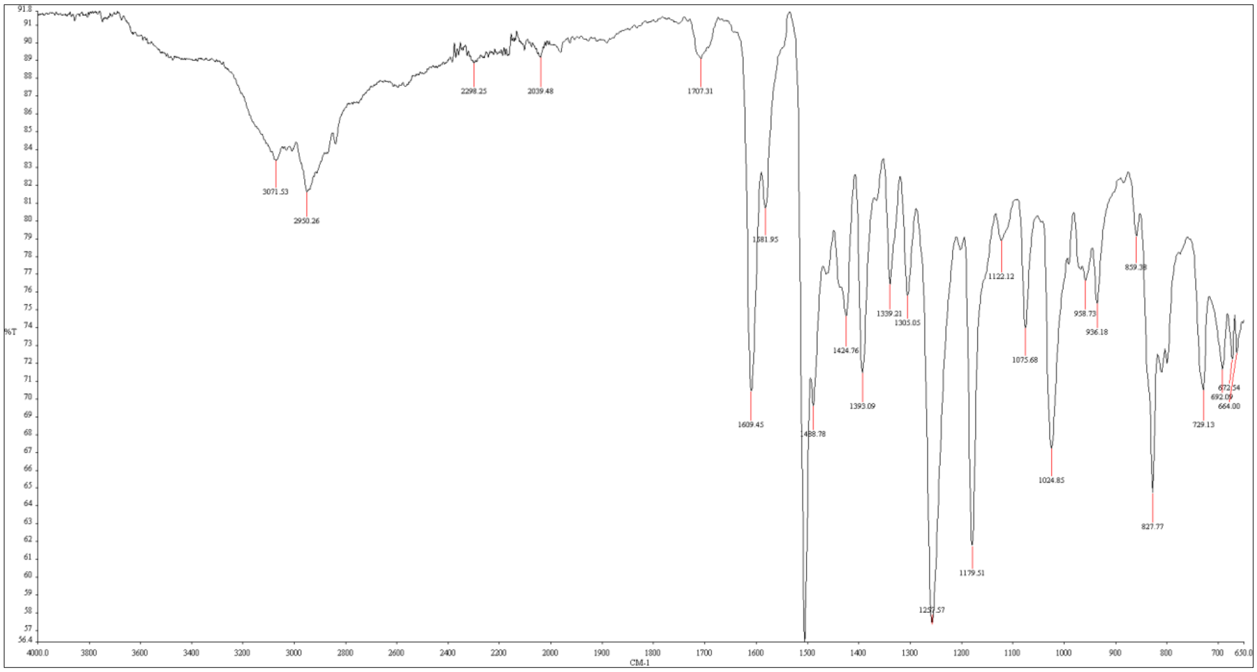

FT-IR of ligand after copper complexation

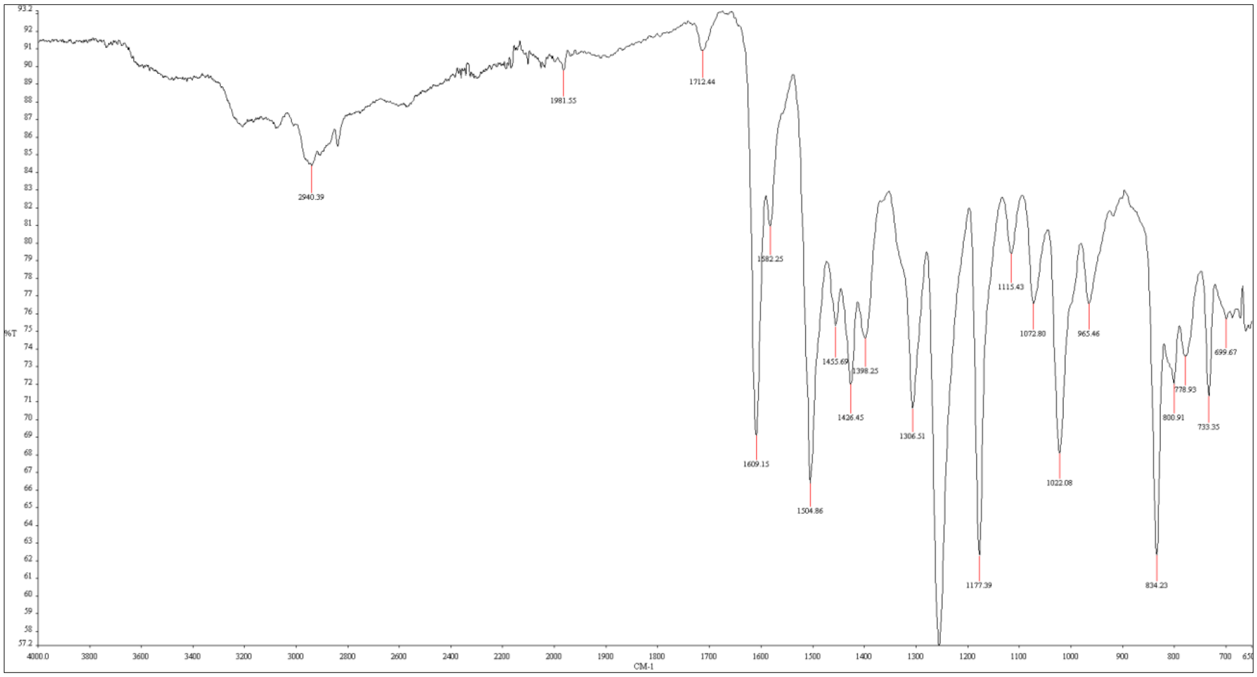

FT-IR of ligand after palladium complexation

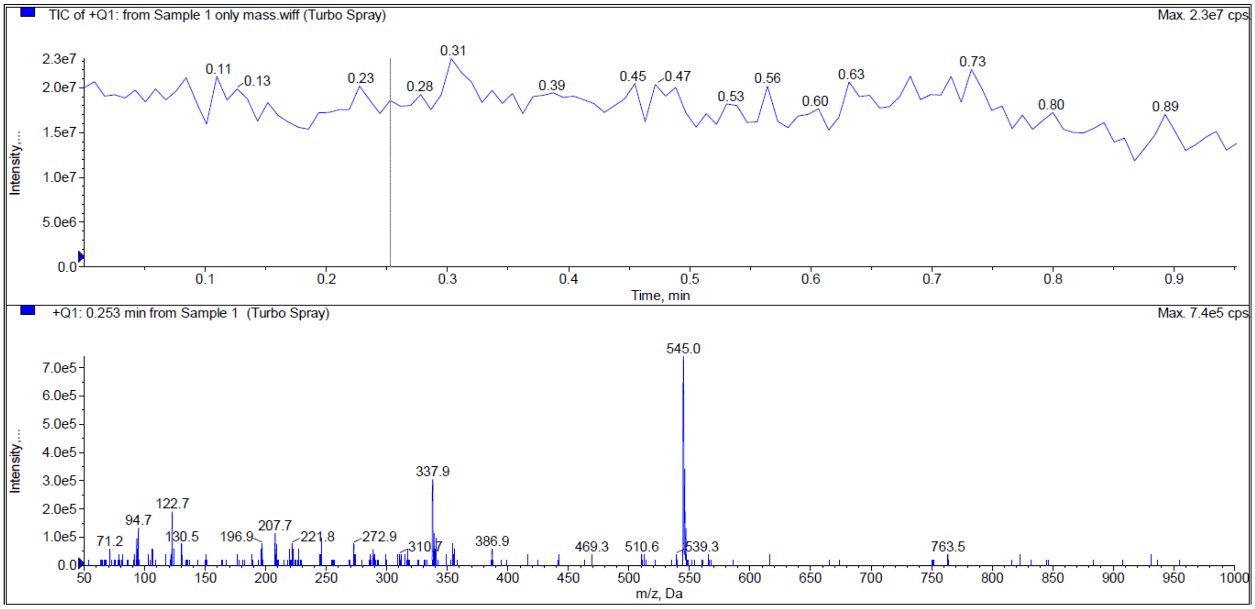

LC-MS Spectrum of target molecule alone

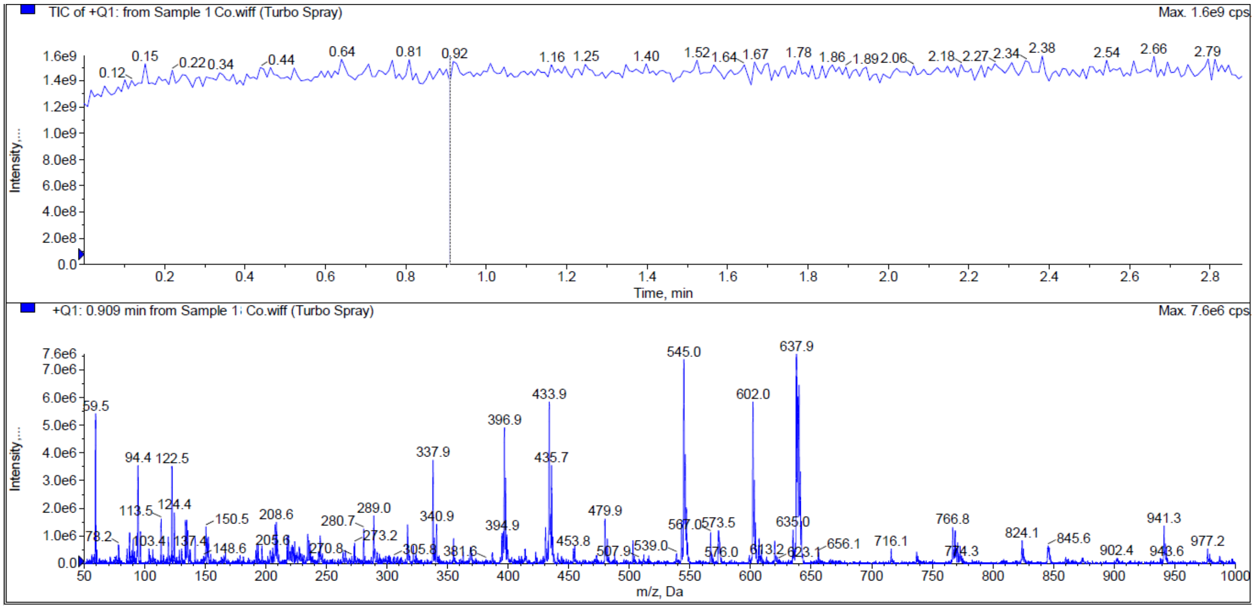

LC-MS Spectrum of target molecule after metal complexation

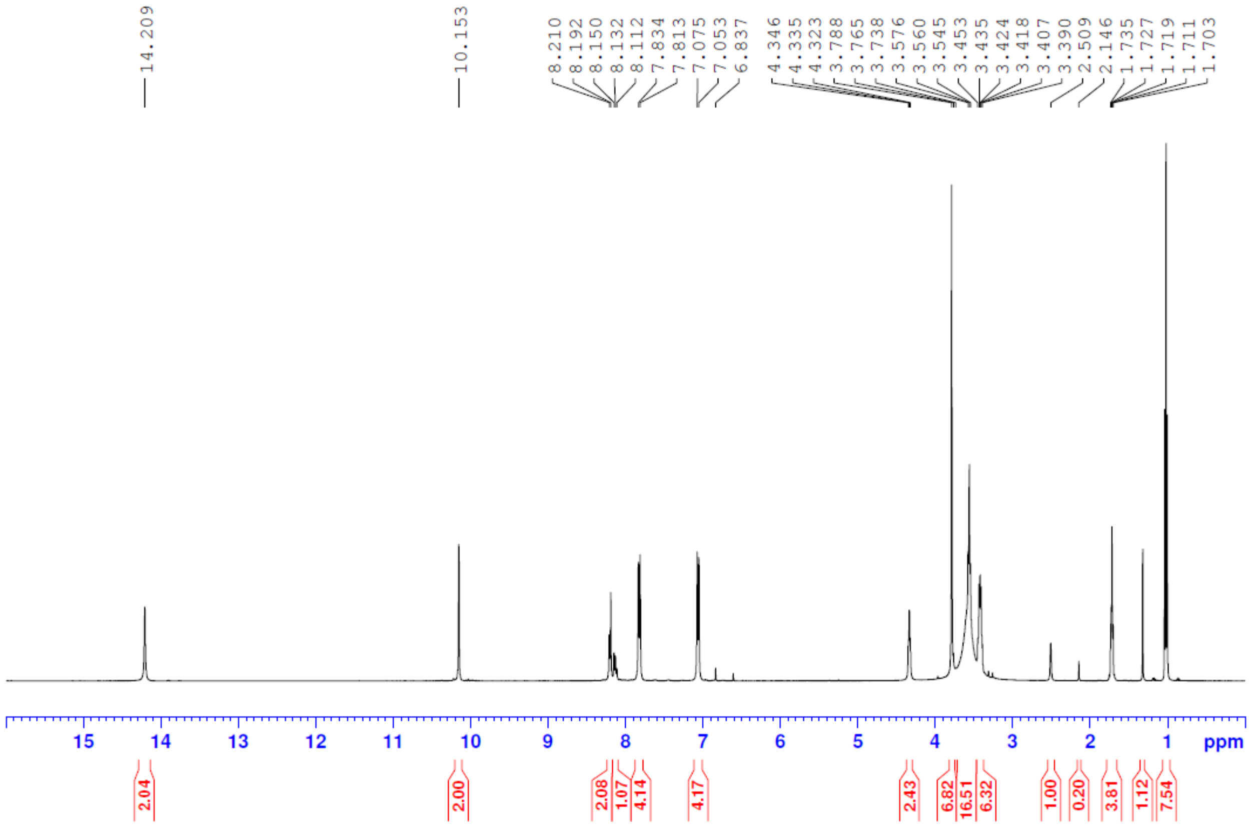

^{1}H NMR of ligand after complexation with cobalt

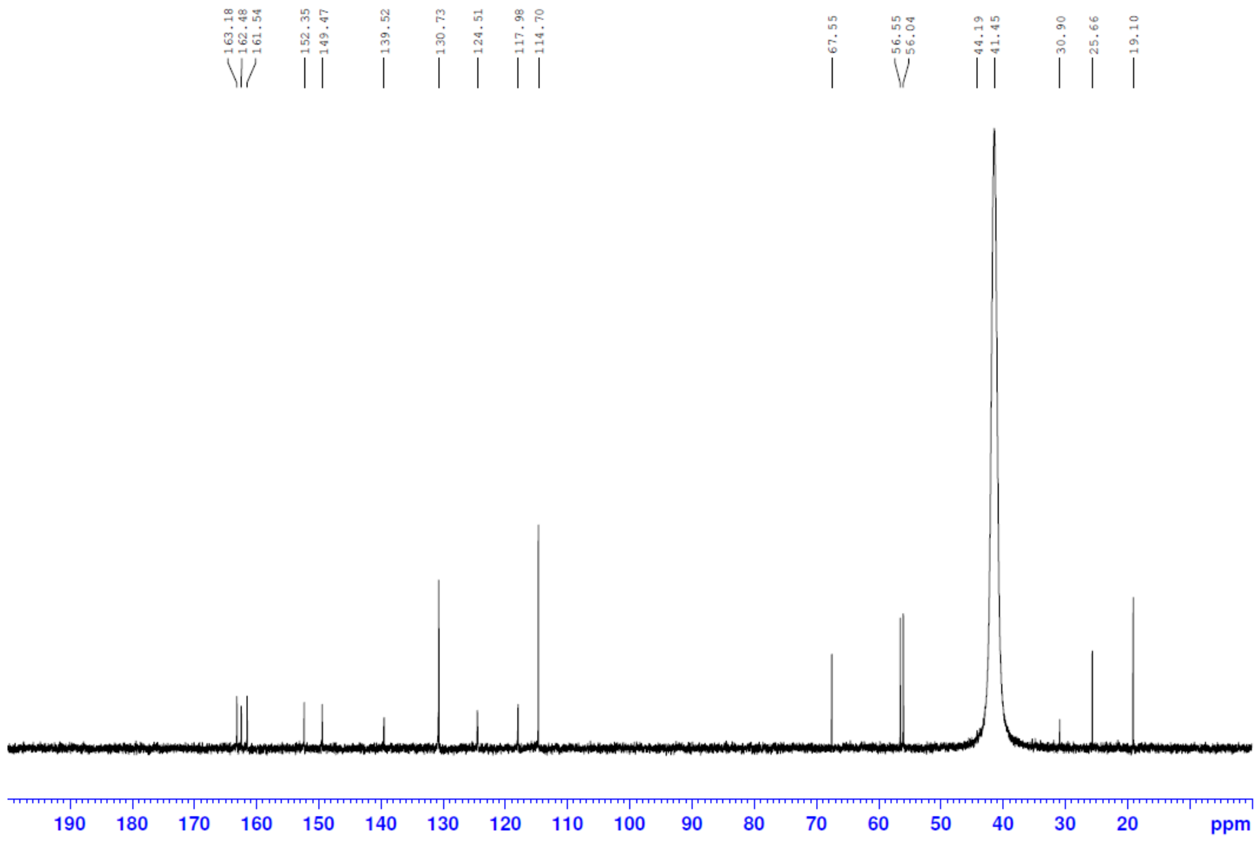

^{13}C NMR of ligand after cobalt complexation

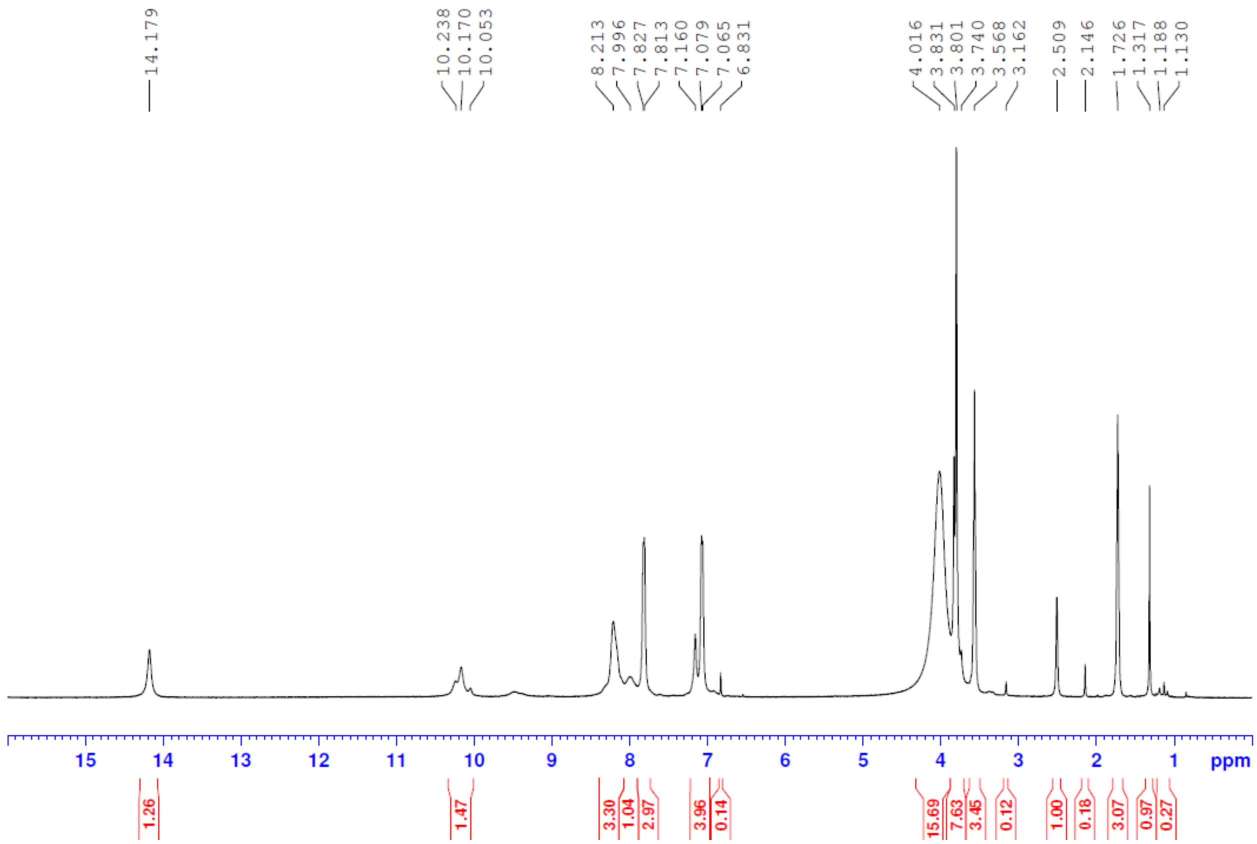

^{1}H NMR of ligand after complexation with copper

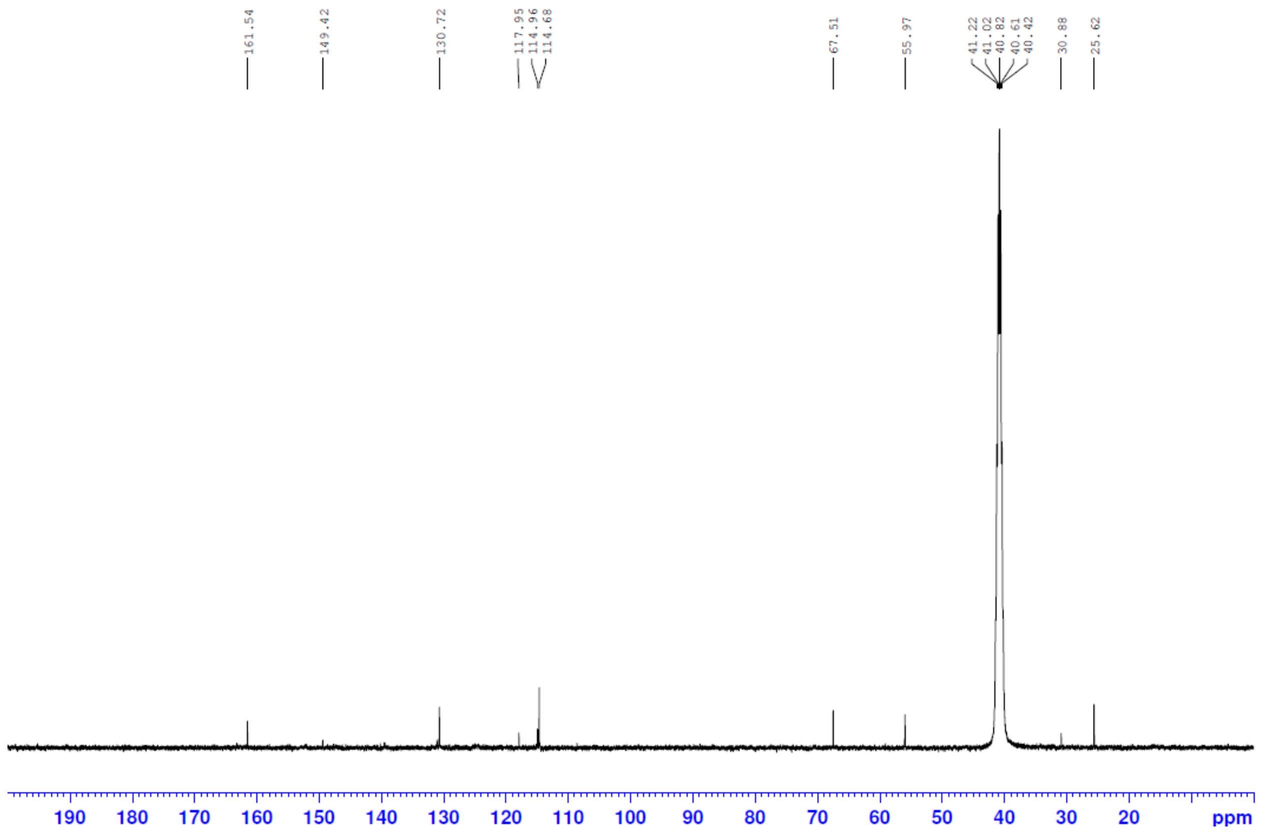

^{13}C NMR of ligand after copper complexation

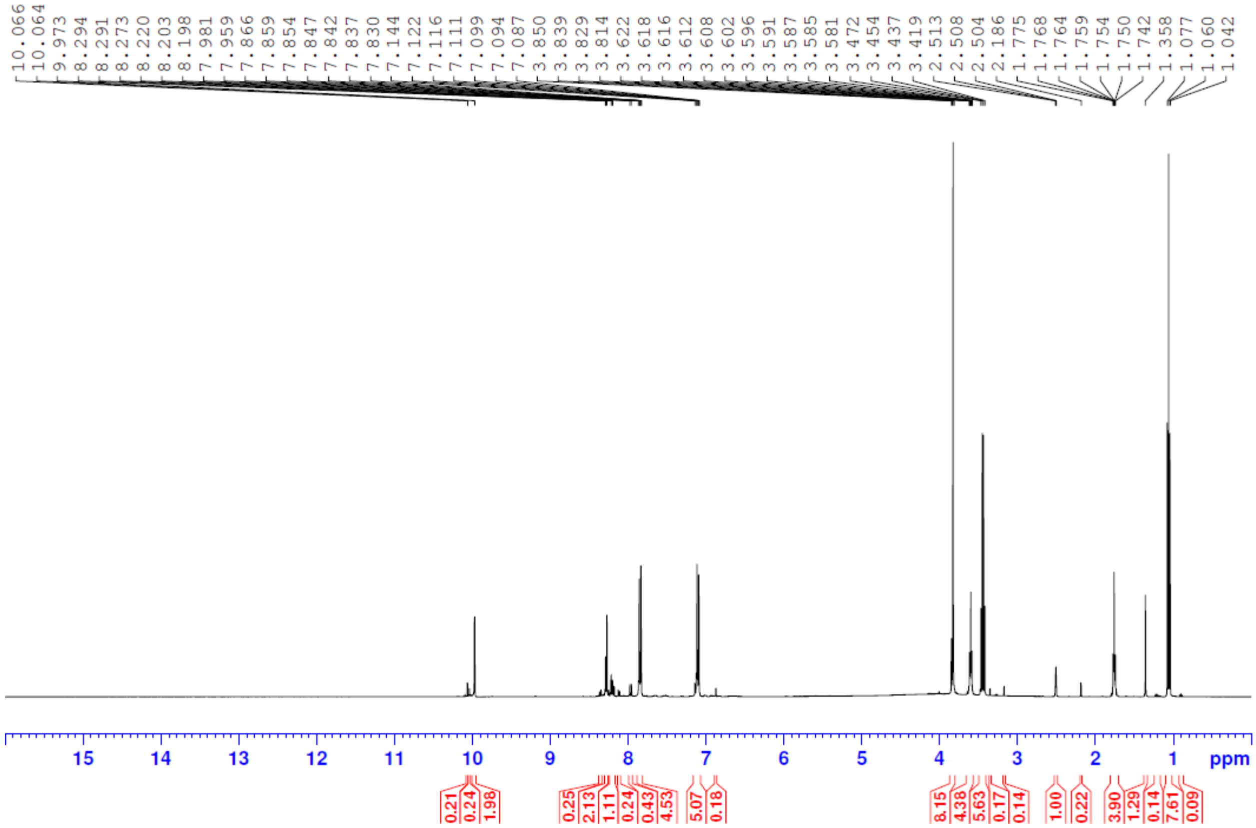

^{1}H NMR of ligand after complexation with palladium

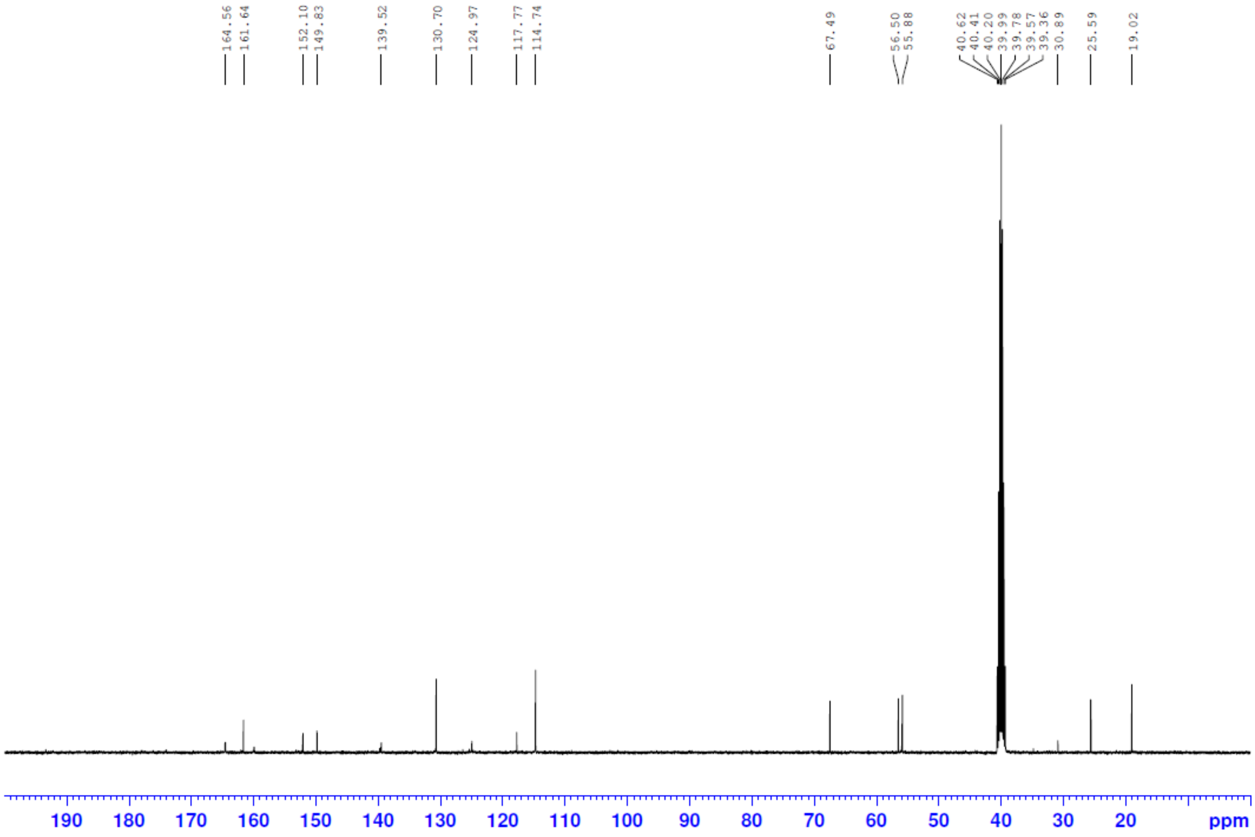

^{13}C NMR of ligand after palladium complexation

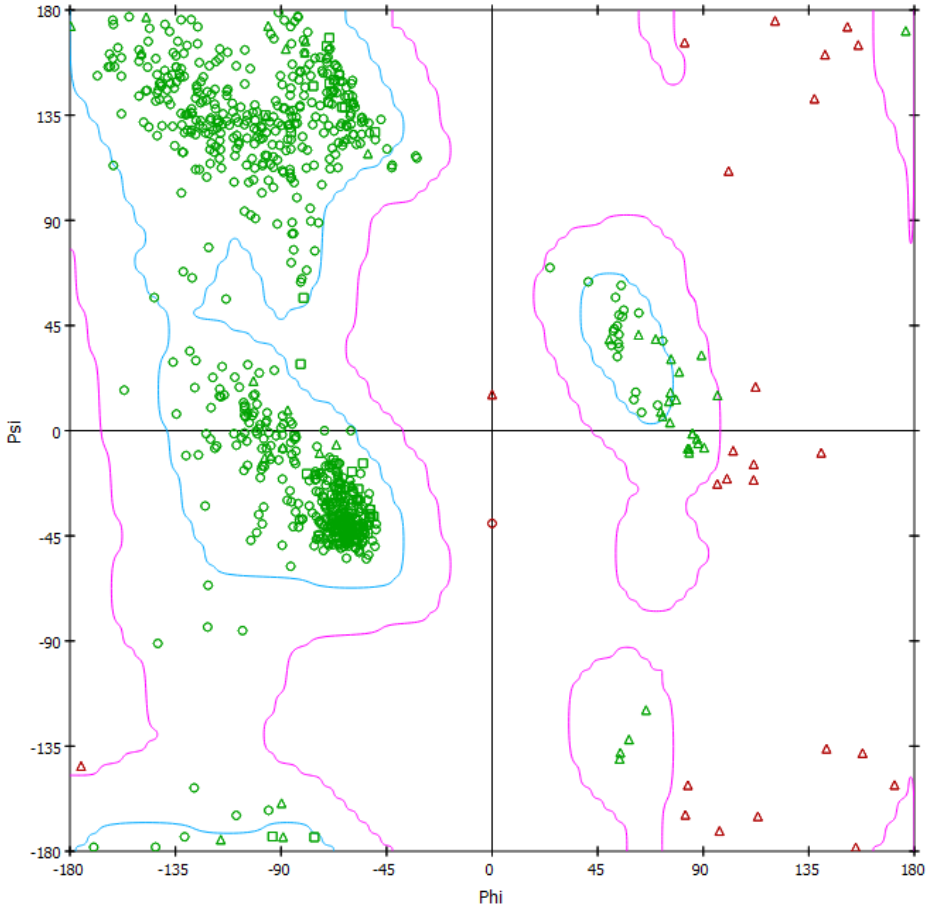

Ramachandran plot of jack bean urease
